# Assessing Molecular Signature for Some Potential Date (*Phoenix dactylifera* L.) Cultivars from Saudi Arabia, Based on Chloroplast DNA Sequences *rpoB* and *psbA-trnH*

**DOI:** 10.3390/ijms12106871

**Published:** 2011-10-17

**Authors:** Fahad Al-Qurainy, Salim Khan, Fahad M. Al-Hemaid, M. Ajmal Ali, M. Tarroum, M. Ashraf

**Affiliations:** 1 Department of Botany and Microbiology, College of Science, King Saud University, Riyadh- 11451, Saudi Arabia; E-Mails: fahad_alqurainy@yahoo.com (F.A.-Q.); fhemaid@ksu.edu.sa (F.M.A.-H.); ajmalpdrc@gmail.com (M.A.A.); med_taroum@yahoo.fr (M.T.); 2 Department of Botany, University of Agriculture, Faisalabad 308400, Pakistan; E-Mail: ashrafbot@yahoo.com

**Keywords:** *Phoenix dactylifera*, dates, molecular signature, Saudi Arabia, *rpoB*, *psbA-trnH*

## Abstract

*Phoenix dactylifera* L. (date palm), being economically very important, is widely cultivated in the Middle East and North Africa, having about 400 different cultivars. Assessment of date cultivars under trading and farming is a widely accepted problem owing to lack of a unique molecular signature for specific date cultivars. In the present study, eight different cultivars of dates viz., *Khodry*, *Khalas*, *Ruthana*, *Sukkari*, *Sefri*, *Segae*, *Ajwa* and *Hilali* were sequenced for *rpoB* and *psbA-trnH* genes and analyzed using bioinformatics tools to establish a cultivar-specific molecular signature. The combined aligned data matrix was of 1147 characters, of which invariable and variable sites were found to be 958 and 173, respectively. The analysis clearly reveals three major groups of these cultivars: (i) *Khodary*, *Sefri*, *Ajwa*, *Ruthana* and *Hilali* (58% BS); (ii) *Sukkari* and *Khalas* (64% BS); and (iii) *Segae.* The economically most important cultivar *Ajwa* showed similarity with *Khodary* and *Sefri* (67% BS).The sequences of the date cultivars generated in the present study showed bootstrap values between 38% and 70% so these sequences could be carefully used as molecular signature for potential date cultivars under trading and selection of genuine cultivars at the seedling stage for farming.

## 1. Introduction

*Phoenix dactylifera* L. (family: *Arecaceae*) which is commonly known as date palm is of considerable economic importance and thus is widely cultivated in the Middle East and North Africa [1]. The Kingdom of Saudi Arabia is the largest producer of dates in the world. There are about 400 different cultivars of dates which are mainly grown in the central, western, and south-western regions of Saudi Arabia [2].

The date palm fruit is a common staple food in the Middle East and North African regions as well as many other tropical and subtropical regions [3]. The most important carbohydrates occurring in large amounts in its fruit are fructose and glucose [4], which are a source of rapid energy for the human body. The quality of dates depends on the amount of dietary fibers they contain, as well as on the presence of essential minerals such as calcium, iron, magnesium, phosphorus, potassium, zinc, selenium and manganese [5]. The fresh and dried dates vary quantitatively and qualitatively in their phenolic acid contents. Besides possessing considerable nutritional value, date fruits are rich in phenolic compounds expressing high antioxidant activity [6]. The extracts of date fruits have been reported to be biologically very active, *i.e.*, it is a potent antioxidant and shows antimutagenic activities [7–11].

Genetically, the date palm is highly diverse due to existence of large number of cultivars distributed in different habitats of Saudi Arabia. A variety of morphological characters of date fruits (viz., shape, size, weight, color, aspects of fruit skin, consistency, texture, *etc*.) and biochemical markers like isozymes and proteins [12–14] have earlier been employed for the identification of date fruits, however, these traits are greatly influenced by environmental factors as well as the developmental stages of the plant. The random amplified polymorphic DNA (RAPD), inter simple sequence repeats (ISSR), and microsatellites have earlier been employed for germplasm characterization of different date palm cultivars from Saudi Arabia and other countries like Egypt, Tunisia and Sudan with similar climatic conditions [15–21]. In most of these studies, considerable genetic diversity has been detected in date palm germplasm using these molecular markers.

Although DNA sequence data and barcoding are now well accepted global standards for species identification [22], they have also some limitations [23]. However, more recently the chloroplast DNA sequences alone or with combination of nuclear sequences are being extensively explored [24–26].

Generally, the chloroplast genome of plants shows maternal inheritance but in a few cases paternal or biparental mode of inheritance has been reported [27,28]. For example, *Passiflora* has been reported to show all three types of chloroplast inheritance [29]. In date palm, maternal inheritance has also been reported while hybridizing the species of genera *Orbignya* and *Phoenix.* Due to putative maternal inheritance of chloroplast DNA it may generate erroneous results in cladistic studies [30].

Since, the proper identification of date cultivars always remains ambiguous, so there is an urgent need of their molecular characterization at least for those potential cultivars which are economically and medicinally important and have high trade value. The rapidly and slowly evolving loci including *psbA-trnH* and *rpoB* differentiate at genera and species level, respectively. To shun perplexing issues due to considerable variation in DNA sequence alignments, the same two locus barcodes were used as alignment associated with the corresponding single-locus barcodes. Moreover, the main reason of choosing these two loci for date palm was that these exhibit considerable genetic variability and divergence, ease of amplification, short sequence length, conserved flanking sites for developing universal primers and ease of alignment and analysis [26,31].

Thus, the main objective of this work was to assess molecular signature of some of the economically and medicinally important date cultivars from Saudi Arabia based on *rpoB* and *psbA-trnH* chloroplast DNA sequences data.

## 2. Results and Discussion

Owing to considerable phenotypic plasticity in *Phoenix* species, it is very difficult to characterize based on vegetative characters. DNA sequence based identification and authentication is a potential method and is being employed for a variety of species. However, sequence variation is an important aspect of barcode identification between species of different genera or of a same genus. Here, the two-loci barcoding system has been employed to overcome the problem faced during the application of a single marker. These two loci *rpoB* and *psbA-trnH* have shown considerable differentiation among the cultivars of date palm as compared to other loci. Both loci exhibited high PCR success with universal primers. We also tried to amplify the *rpoC* and ITS regions, however, these loci were not amplified successfully (data not shown here). The combined length of the *rpoB* and *psbA-trnH* from all date palm cultivars examined in the present study ranged from 1137 to 1143 bp with *rpoB* of 463–469 bp and *psbA-trnH* of 674 bp. The plastome genomes are highly AT-rich and average being around 63%. However, the GC content in *rpoB* varied from 39 to 41%. The total length of *psbA-trnH* was found constant in all the samples; however, the GC content varied from 28 to 32% (Table 1). The percent GC content variation was more in *rpoB* locus because of its coding property while *psbA-trnH* is non-coding. The length variation was found more in *rpoB* spacer sequence while it was fixed in *psbA-trnH*. The reason of variation in length of *rpoB* may be due to the genomic rearrangement of the inverted repeat [32].

Insertions or deletions (indels) were necessary to align the sequences. The combined aligned data matrix were of 1147 characters of which invariable (monomorphic) sites were 958, variable (polymorphic) sites 173, out of which parsimony informative sites were 67. The indels ranged in length from 1 to 10 bp (see supplementary).

The polymorphism among the data set was noted. The total number of polymorphic sites, variance of haplotype diversity, nucleotide diversity (Pi), theta (per site) from Eta and average number of nucleotide differences (K) among all cultivars was found to be 135, 0.00391, 0.0686, 0.0686, 45.893, respectively in *psbA-trnH*, while 33, 0.0339, 0.0276, 0.0276, 11.321, respectively, in *rpoB* (Table 2). The *psbA-trnH* showed more polymorphic sites than the *rpoB* locus; hence, it was more informative and proved very effective in differencing the date palm cultivars. Moreover, *psbA-trnH* has been used successfully in angiosperms, but now further research is required to verify its efficacy on mosses, ferns and gymnosperms [31].

The genetic divergence evaluated among the date palm cultivars may be due to the dispersal of off-shoots, pollen grains and seeds, *etc*. Off-shoots and pollen grains are extensively distributed among farmers within a village, province or country, while seed dispersal takes place by other means such as travelers and traders across geographic borders [21]. The other reasons of sequence variability among the date cultivars could be mountains and low lands, different matting, mutation, genetic drift, gene flow, selection, long-term evolution history, successional stages, and human activities are some of the promising factors that affect genetic variation patterns among plant populations [33–35]. Furthermore, environmental factors and heterogeneous forms of cultivars may also be one of the reasons of variability [36]. The dioecious nature of date palms may also be one of the reasons for its high genetic variability as has been observed in Tunisian date palms [34].

**Figure 1:**
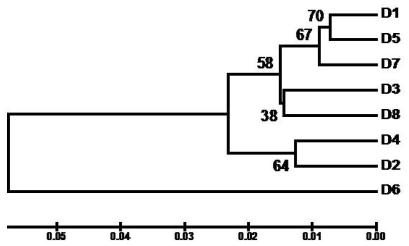
Unweighted Pair Group Method with Arithmetic Mean (UPGMA) tree inferred from combined data set of *rpoB* and *psbA-trnH* DNA sequences showing relationships among the date palm cultivars. The percentages of replicate trees in which the associated cultivars clustered together in the bootstrap test (100 replicates) are shown next to the branches.

Relationships among the date palm cultivars evaluated in the present study were inferred using the Unweighted Pair Group Method with Arithmetic Mean (UPGMA) [37], which reveals the optimal tree with the sum of branch length, 0.1965 (Figure 1). The analysis clearly reveals three major clades: (i) the cultivars *Khodary*, *Sefri*, *Ajwa*, *Ruthana* and *Hilali* clustered together with 58% bootstrap support; (ii) the cultivars *Sukkari* and *Khalas* are clustered together with 64% bootstrap support; and (iii) the sequence pattern of *Segae* was found different from the other cultivars. It is important to note that the most economically important cultivar *Ajwa* showed clading with *Khodary* and *Sefri* (67% bootstrap support). All cultivars which were morphologically different to each other also differed in sequence up to some extent at both loci and thus each sequence would act as a molecular signature for each cultivar.

The phylogenetic relationships among the date palm cultivars have been also evaluated in different countries viz., Tunisia, California and Morocco using various molecular markers such as ISSR, AFLP and RAPD [17,21,38–40]; microsatellite [20,21]; and isozyme [14,41]. These markers showed high polymorphism among the date palm cultivars, but it remained difficult to effectively characterize them. Therefore, the chloroplast spacer sequences were employed for the discrimination of various cultivars of date palm. These markers generally show better phylogenetic relationships than do the nuclear DNA markers. However, the utility of chloroplast spacer sequences including *rpoB* and *psbA-trnH* gene sequences in molecular typing has earlier been well reviewed [24–26,31,42–48]. The sequences of the date palm cultivars generated in the present study could be used as molecular signature for specific cultivars of dates under trading and selection of genuine cultivars at the seedling stage under farming.

## 3. Materials and Methods

### 3.1. Sampling

The fruits of different cultivars of date palm viz., *Khodry*, *Khalas*, *Ruthana*, *Sukkari*, *Sefri*, *Segae*, *Ajwa*, and *Hilali* were purchased (in triplicate) from a commercial market in Riyadh, Saudi Arabia (Table 1). The seeds were taken out from the fruits and then washed with sterilized distilled water and thereafter, disinfected with 0.01% NaOCl. The seeds were again washed with sterilized distilled water to remove extra NaOCl from the seed surface. Further, seeds were soaked in sterilized water for four weeks to attain proper germination. The germinated seeds of all cultivars were used for DNA extraction.

### 3.2. Genomic DNA Extraction

#### 3.2.1. Procedure

Genomic DNA was isolated from the germinated seeds of all date palm cultivars using a modified CTAB (cetyl trimethyl ammonium bromide) method [49]. A proportion (0.01 g) seedling was ground in 800 μL of extraction buffer (100 mM Tris buffer pH 8, 25 mM EDTA, 2 M NaCl, 3% CTAB and 3% polyvinyl pyrrolidone). The resultant seedling paste was transferred to a microcentrifuge tube and incubated at 65 °C for 20 min with frequent mixing. The mixture was then cooled to room temperature and an equal volume of chloroform: isoamyl alcohol (24:1) was added to it. The mixture was centrifuged at 12000 rpm for 10 min. The clear upper aqueous part was then transferred to a new microfuge tube, added a 2/3 volume of ice-cooled isopropanol to it and incubated the whole mixture at −20 °C for 2 h. For the collection of nucleic acid, the mixture was centrifuged at 1000 rpm for 10 min. The resulting pellet was washed twice with 80% ethanol and then air-dried under a sterile laminar hood. The nucleic acid was dissolved in sterilized distilled water at room temperature and the contaminating RNA was removed by treating with RNase A (10 mg/mL) for 30 min at 37 °C. DNA concentration and purity were determined by measuring the absorbance of diluted DNA solution at 260 nm and 280 nm. Further, the quality of the DNA was determined using agarose gel electrophoresis staining with ethidium bromide.

#### 3.2.2. Amplification and Sequencing

The *rpoB* and *psbA-trnH* sequences were amplified using the AccuPower HF PCR PreMix (Bioneer, Daejeon, South Korea) in 20 μL volumes containing 2 μL of 10× buffer, 300 μM dNTPs, 1 μL of a 10 pM solution of each primer, 1 unit of HF DNA polymerase (see Table 3 for primer sequence and reaction conditions).

The amplified PCR products were checked on 1.2% agarose gel containing ethidium bromide. The size was confirmed using DNA molecular weight marker. The PCR products were purified with the SolGent PCR Purification Kit-Ultra (SolGent, Daejeon, South Korea) prior to sequencing. The sequencing reaction was performed with the BigDye Terminator cycle sequencing kit (Perkin-Elmer, Applied Biosystems) by following the manufacturer’s instructions in an ABI PRISM 3730XL DNA Analyzer (Perkin-Elmer, Applied Biosystems). Each sample was sequenced in the sense and antisense directions and analyzed with ABI Sequence Navigator software (Perkin-Elmer/Applied Biosystems).

### 3.3. DNA Sequence Data Analysis

Sequence alignment was performed using the ClustalX version 1.81 [50]. The aligned sequences were then subsequently adjusted manually using BioEdit [51]. Insertion-deletions (Indels) were scored as single characters when we had confidence in positional homology. The boundaries of each sequence were determined by comparing them with earlier published sequences available in GenBank- taxonomy database of National Centre for Biotechnology Information (www.ncbi.nlm.nih.gov/GenBank). Gaps were treated as missing data in phylogenetic analyses. All sequences generated in the present study were deposited in GenBank for reference and accession numbers are given in (Table 1). Nucleotide polymorphism among the cultivars was analyzed using the software DnaSP v4.5 [52]. The cladistic analysis of aligned sequences was performed using phylogenetic analysis software MEGA4 [53] following the UPGMA method [37]. The Maximum Composite Likelihood method [54] was used to compute the evolutionary distances among the date palm cultivars, which were in the units of the number of base substitutions per site. Codon positions included were 1st + 2nd + 3rd + Noncoding. All positions containing gaps and missing data were removed from the dataset (Complete deletion option). The final data set contained a total of 1131 positions.

## 4. Conclusion

Identification and authentication is very important to maintain the quality and efficacy of cultivars of dates in local markets. Some cultivars are being used in drug formulations, for which their proper identification is very important for evaluation of their potential medicinal efficacy. The sequences of the date palm cultivars generated in the present study show bootstrap values range from 38–70%, which is not very high, so these sequences could be used with some caution as molecular signature for specific cultivars of dates under trading and selection of genuine cultivars at the seedling stage under farming.

## Figures and Tables

**Table 1:** Sequence characteristics of the date palm cultivars examined in the present study.

Cultivars	Abbreviation	Sequence characteristics					

		*rpoB*			*psbA-trnH*		

		Accession No.	Total length	%GC	Accession No.	Total length	%GC
*Khodry*	D1	JN854236	463	39	JN854228	674	28
*Khalas*	D2	JN854237	464	40	JN854229	674	29
*Ruthana*	D3	JN854238	463	39	JN854230	674	29
*Sukkari*	D4	JN854239	469	41	JN854231	674	29
*Sefri*	D5	JN854240	464	39	JN854232	674	29
*Segae*	D6	JN854241	467	40	JN854233	674	32
*Ajwa*	D7	JN854242	463	39	JN854234	674	29
*Hilali*	D8	JN854243	463	39	JN854235	674	29

**Table 2:** Sequence polymorphism among the date palm cultivars.

Sequence polymorphism	*psbA-trnH*	*rpoB*
Number of polymorphic sites	135	33
Variance of haplotype diversity	0.00391	0.0339
Nucleotide diversity (Pi)	0.0686	0.0276
Theta (per site) from Eta	0.0807	0.0276
Average number of nucleotide differences (K)	45.893	11.321

**Table 3:** Primer sequence and reaction conditions for PCR amplification.

Gene	Primer	Primer sequence 5′-3′	Reaction conditions
*psbA-trnH*	Forward	GTTATGCATGAACGTAATGCTC	94 °C 1 min, 94 °C 30 s, 53 °C 40 s, 72 °C 30 s, 40 cycles, 72 °C 5 min
	Reverse	CGCGCATGGTGGATTCACAAATC	
*rpoB*	Forward	ATGCAACGTCAAGCAGTTCC	94 °C 1 min, 94 °C 30 s, 53 °C 40 s, 72 °C 30 s, 40 cycles, 72 °C 5 min
	Reverse	GATCCCAGCATCACAATTCC	
